# The Permeability Transition in Plant Mitochondria: The Missing Link

**DOI:** 10.3389/fpls.2015.01120

**Published:** 2015-12-15

**Authors:** Marco Zancani, Valentino Casolo, Elisa Petrussa, Carlo Peresson, Sonia Patui, Alberto Bertolini, Valentina De Col, Enrico Braidot, Francesco Boscutti, Angelo Vianello

**Affiliations:** Plant Biology Unit, Department of Agricultural and Environmental Sciences, University of UdineUdine, Italy

**Keywords:** permeability transition, plant mitochondria, ATP synthase, exaptation, environmental stress

## Abstract

The synthesis of ATP in mitochondria is dependent on a low permeability of the inner membrane. Nevertheless, mitochondria can undergo an increased permeability to solutes, named permeability transition (PT) that is mediated by a permeability transition pore (PTP). PTP opening requires matrix Ca^2+^ and leads to mitochondrial swelling and release of intramembrane space proteins (e.g., cytochrome *c*). This feature has been initially observed in mammalian mitochondria and tentatively attributed to some components present either in the outer or inner membrane. Recent works on mammalian mitochondria point to mitochondrial ATP synthase dimers as physical basis for PT, a finding that has been substantiated in yeast and *Drosophila* mitochondria. In plant mitochondria, swelling and release of proteins have been linked to programmed cell death, but in isolated mitochondria PT has been observed in only a few cases and in plant cell cultures only indirect evidence is available. The possibility that mitochondrial ATP synthase dimers could function as PTP also in plants is discussed here on the basis of the current evidence. Finally, a hypothetical explanation for the origin of PTP is provided in the framework of molecular exaptation.

## The Permeability Transition

ATP synthesis in mitochondria occurs by a chemiosmotic coupling of substrate oxidation and phosphorylation (Mitchell, 1961). This explanation is based on the highly selective permeability of the inner mitochondrial membrane (IMM) and on utilization of protonmotive force by the F_1_F_O_ ATP synthase (F-ATPase) for the synthesis of ATP. Nevertheless, a sudden increase in permeability of the IMM has been described in the 1950s (Raaflaub, 1953a,b) and characterized in the late 1970s (Haworth and Hunter, 1979; Hunter and Haworth, 1979a,b). Initially considered an artifact, later it has been named Permeability Transition (PT) and associated to a pore, the Permeability Transition Pore (PTP). The appreciation of its relevance has increased since it has been related to many diseases in mammals, including reperfusion injury of the heart and muscular dystrophy (Bernardi, 2013a). This mitochondrial PT requires matrix Ca^2+^ and is favored by matrix P_i_, as well as benzodiazepine Bz-423 and thiol oxidants, while it can be inhibited by Mg^2+^, thiol reductants, ADP and ATP (Bernardi, 2013b). Cyclosporin A (CsA) acts as inhibitor of PT (Crompton et al., 1988) by binding with the peptidyl-prolyl isomerase Cyclophilin D (CyPD) (Halestrap and Davidson, 1990). The features of PTP (e.g., pore diameter of ∼2.8 nm and size exclusion of about 1500 Da) are consistent with those described for the Mitochondrial Mega-Channel (MMC), a high-conductance channel, which is considered to be its electrophysiological equivalent (Szabó and Zoratti, 1992).

## The PT in Plants

The first evidence of a Ca^2+^-induced and CsA-delayed collapse of transmembrane electrical potential difference (ΔΨ) in pea stem mitochondria dates back to 1995 (Vianello et al., 1995). PT has been then observed in different plant species, although the features of this phenomenon cannot be summarized in a straightforward model (**Table 1**). Potato tuber mitochondria exhibit a typical Ca^2+^/P_i_-induced PT, inhibited (Arpagaus et al., 2002) or not (Fortes et al., 2001) by CsA. These mitochondria do not show any Ca^2+^ uptake, suggesting an external effect of Ca^2+^ on PT (Fortes et al., 2001), which is not consistent with the observations in mammals (Bernardi et al., 2015). The PT described in oat leaves (Curtis and Wolpert, 2002) and wheat roots (Virolainen et al., 2002) shows a Ca^2+^/P_i_ -induced ΔΨ collapse and matrix swelling, which are CsA-insensitive. Calcium uptake by isolated plant mitochondria occurs spontaneously in wheat, but requires the addition of the Ca^2+^/H^+^ ionophore A23187 in oat.

**Table 1:** Characteristics of permeability transition (PT) in plant mitochondria.

Plant material	Ca^2+^ stimulation	CsA inhibition	Sucrose swelling	Cytochrome *c* release	Reference
Etiolated pea stem	Yes	Yes	No	Not detected	Vianello et al., 1995
Potato tuber	Yes (external)	No	Yes	Yes	Fortes et al., 2001
Potato tuber	Yes	Yes	Yes	Yes	Arpagaus et al., 2002
Oat leaves	Yes (with A23187)	No	Yes	Yes	Curtis and Wolpert, 2002
Wheat roots	Yes	No	Yes	Yes	Virolainen et al., 2002

Indirect evidence of PT in plants has been also based on the CsA-induced inhibition of programmed cell death (PCD), reviewed by Vianello et al. (2007, 2012). However, the prevention of PCD might depend on CsA binding to cytosolic Cyclophilin A (a ubiquitous enzyme) that drives enzymatic cascades (Lu et al., 2007), linked to oxidative stress (Nigro et al., 2013).

## The Mitochondrial Ca^2+^ Accumulation in Plants

The PT requires Ca^2+^ accumulation into the mitochondrial matrix (i.e., matrix Ca^2+^ is a permissive factor, although it may not be sufficient *per se*). Calcium transport in isolated plant mitochondria exhibits distinct features. The uptake could be mediated by a low-affinity electrophoretic P_i_-dependent symport, with low or no sensitivity to ruthenium red and lanthanides (Dieter and Marme, 1980; Akerman and Moore, 1983; Silva et al., 1992), but also by a uniport mechanism (Zottini and Zannoni, 1993). CsA inhibits mitochondrial Ca^2+^ transport in *Citrus* (de Oliveira et al., 2007), suggesting its synergic effect with PT. A low concentration of matrix free Ca^2+^ (∼100 nM) is maintained under steady state, where influx is balanced by an eﬄux through a yet speculative Na^+^-independent Ca^2+^/H^+^ antiport mechanism (Nomura and Shiina, 2014). The influx of Ca^2+^ in plant mitochondria is highly variable, depending on species and tissues, or might be even completely absent (Martins and Vercesi, 1985). *In vivo* Ca^2+^ dynamics have been monitored by fluorescent probes targeted to plant mitochondria (Manzoor et al., 2012; Loro and Costa, 2013). Matrix Ca^2+^ uptake can be induced by abiotic stresses such as heat, oxidative stress, or anoxia, and follows the cytosolic Ca^2+^ pattern (Subbaiah et al., 1998; Logan and Knight, 2003; Schwarzländer et al., 2012; Rikhvanov et al., 2014).

Homologue genes of mammalian mitochondrial Ca^2+^ uniporter (MCU) and its regulatory protein MICU1 have been found in plants (Bick et al., 2012; Stael et al., 2012; Rikhvanov et al., 2014). The MICU1 homologue in *Arabidopsis* (AtMICU) is a negative regulator of mitochondrial Ca^2+^ uptake in root tips, providing strong evidence for the operation of a mitochondrial Ca^2+^ uniporter in plants (Wagner et al., 2015).

## The Involvement of PT/PCD in Plant Development and Stress Responses

The physiological role of mitochondrial PT in plants is often related to developmental processes (Reape et al., 2015) and mild environmental stresses, which involve also PCD in many cases. However, the mechanistic link between PT and PCD remains still speculative.

Permeability transition/programmed cell death are fundamental in the selection of damaged cells and in sculpturing new anatomical and morphological structures (Van Hautegem et al., 2015). Morphological modifications are also needed for adaptive responses to environment (e.g., climate changes) and, more in general, for fitness increase. In particular, *Aponogeton madagascariensis* forms lacunae on its leaves by executing PCD, which is inhibited by CsA, suggesting the involvement of PT (Lord et al., 2013). In aerenchyma formation, lack of oxygen induces stress characterized by mitochondrial PT, ATP depletion, and PCD induction (Yamauchi et al., 2013). Consistently, stressed pea plants show cytochrome *c* release, followed by DNA fragmentation (Sarkar and Gladish, 2012).

Programmed cell death is a common response in plants subjected to abiotic and biotic stresses, which may be linked to the sessile lifestyle, providing a survival strategy for the whole organism. Excess of UV-C stimulates reactive oxygen species (ROS) formation and collapse of ΔΨ in *Arabidopsis* mitochondria (Gao et al., 2008). The role of PT has also been described in case of extreme temperatures. In *Arabidopsis* protoplasts, heat stress induces mitochondrial swelling, and ΔΨ loss, but these damages are counteracted by a heat shock transcription factor (Zhang et al., 2009). Similarly, ROS and mild heat shock induce mitochondrial PT and the subsequent induction of cell death in *Arabidopsis* protoplasts, which are prevented by the superoxide dismutase analog TEMPOL, by the Ca^2+^ channel-blocker lanthanum chloride, and by CsA (Scott and Logan, 2008). The role of mitochondria in PCD is confirmed in heat-stressed rice protoplasts, where mHSP70 overexpression maintains mitochondrial ΔΨ, partially inhibits cytochrome *c* release and suppresses PCD by lowering ROS formation (Qi et al., 2011). In wheat cells subjected to freezing, ROS-dependent PCD is associated to ΔΨ collapse and cytochrome *c* release (Lyubushkina et al., 2014). In salt-stressed tobacco protoplasts, PCD is triggered by ROS produced by mitochondria, through a process controlled by a CsA-sensitive PT (Lin et al., 2006).

The response to heavy metals requires the participation of mitochondrial PT. In particular, aluminum triggers a high ROS production in peanut, by plasmalemma NADPH oxidases, which induce mitochondrial mediated-PCD (Huang et al., 2014). Consistently, metal phytotoxicity appears to be also mediated by PT in aluminum- treated *Arabidopsis* protoplasts (Li and Xing, 2011) and in cadmium-treated rice roots (Yeh et al., 2007).

Biotic stress, such as pathogen attack, may lead to protoplast shrinkage, mitochondria swelling and cytochrome *c* release. These responses appear to be associated to PCD involvement during the hypersensitive response, a strategy to counteract biotrophic pathogens. The generation of a defensive layer, promoted by PT-induced PCD, has been shown in *Arabidopsis.* In particular, PCD is mediated by a rapid decrease in mitochondrial ΔΨ, which is partially counteracted by CsA (Yao et al., 2004). Finally, there is evidence on the release of cytochrome *c* induced by elicitors such as harpin or victorin (Curtis and Wolpert, 2002; Krause and Durner, 2004).

## The Molecular Structure of PTP

The components involved in PTP formation initially included the voltage-dependent anion channel, the benzodiazepine receptor, the adenine nucleotide translocase and the phosphate carrier. This model has been questioned, since isolated mitochondria from organisms where the expression of each of these proteins has been suppressed still exhibit a PT (Kokoszka et al., 2004; Krauskopf et al., 2006; Baines et al., 2007; Gutiérrez-Aguilar et al., 2014; Šileikytė et al., 2014).

Recent evidence shows that F-ATPase is involved in PTP formation in different species and *taxa* (Bernardi, 2013b; Bonora et al., 2013; Alavian et al., 2014). This enzyme is highly conserved in both prokaryotes and eukaryotes (Hamasur and Glaser, 1992; Heazlewood et al., 2003), consisting in the hydrophilic F_1_ and the hydrophobic F_O_ sectors, which operate in concert to carry out distinct functions (Antoniel et al., 2014).

The F_1_ contains five subunits: α and β forming the catalytic region, while γ, δ, and ε are organized in the central stalk. In all eukaryotes these subunits show a high degree of similarity in the sequences (Hamasur and Glaser, 1992; Antoniel et al., 2014; Jiko et al., 2015), while the subunit composition of the F_O_ varies among different *taxa* and species (Hamasur and Glaser, 1992). For details about F-ATPase components in mammals, fungi and algae, see Vázquez-Acevedo et al. (2006), van Lis et al. (2007), Dabbeni-Sala et al. (2012), Antoniel et al. (2014), Lee et al. (2015) and Liu et al. (2015). Specific subunits have been characterized in plants such as sweet potato (Morikami et al., 1992), potato (Dell’Orto et al., 1993; Polgreen et al., 1995) and soybean (Smith et al., 1994).

Plant F_1_ includes the classical five-subunit structure (Hamasur and Glaser, 1990, 1992), and also a 24 kDa protein (Li et al., 2012), but the picture of F_O_ components remains still incomplete. Several proteins belonging to F_O_ have been identified in spinach (Hamasur and Glaser, 1992), potato (Jänsch et al., 1996), rice (Heazlewood et al., 2003), and *Arabidopsis* (Heazlewood et al., 2003; Meyer et al., 2008; Klodmann et al., 2011). As shown by Klodmann et al. (2011) and by Li et al. (2012), F_O_ includes subunits a, c, d, 4 (corresponding to subunit b or orf25, Heazlewood et al., 2003), a 6 kDa protein (plant specific), subunit 8 (also called AL6 or orfB, Heazlewood et al., 2003), ATP17 (plant specific) and Oligomycin Sensitivity-Conferring Protein (OSCP), sometimes referred to as δ’ in plants (Morikami et al., 1992), for some authors belonging to F_1_ (Jänsch et al., 1996). Subunit g was found detached from F-ATPase monomer, suggesting that it could represent a dimer-specific protein (Meyer et al., 2008; Klodmann et al., 2011). Plant subunit e sequences have been identified so far only in protein databases for few species (e.g., rice and *Medicago truncatula*).

Multimeric structures of F-ATPase are present in animal, fungi (Davies et al., 2011; Seelert and Dencher, 2011; Liu et al., 2015) and plant mitochondria (Eubel et al., 2003, 2004; Krause et al., 2004; Bultema et al., 2009). Eubel et al. (2003) highlighted the presence of F-ATPase dimers in *Arabidopsis*, potato, bean, and barley. The relative abundance of dimers in plants is low, with respect to the total F-ATPase, and even lower when comparing different organisms (Eubel et al., 2003, 2004).

Rows of F-ATPase dimers in *cristae* seem to be a universal feature of all mitochondria (Davies et al., 2011) that enable the formation of highly curved ridges in *cristae* (Davies et al., 2012). The Inhibitory factor 1 (IF_1_) that binds F-ATPase at low pH (Campanella et al., 2008) could favor dimer formation even if it is not clear how it improves dimer stability. The arrangement of F-ATPase in mammals and fungi is different from that of potato, being the angle between monomers in the latter larger (∼115°) than in the former (∼80°) (Davies et al., 2011). Interestingly, this correlates with *cristae* morphology observed for many plant mitochondria, where irregular saccular structures with a less convex curvature appear particularly prevalent (Douce, 1985). In aging *Podospora anserina* (*Ascomycetes*) mitochondria, the IMM is progressively vesiculated, the *cristae* collapse and the F-ATPase dimers are disassembled (Daum et al., 2013). The impairment of ATP synthesis, and the outer membrane rupture by swelling, lead to the release of pro-apoptotic factors and, finally, to cell death.

Animal mitochondria F-ATPase dimers have been shown to act as pores with properties of the PTP (Giorgio et al., 2013). CyPD modulates F-ATPase activity by binding OSCP (Giorgio et al., 2009) and this interaction is favored by P_i_, while CsA displaces CyPD from the enzyme. F-ATPase is inhibited by Bz-423, which binds to OSCP (Cleary et al., 2007). These features are consistent with those observed for PT regulation. Magnesium, Ca^2+^, adenine nucleotides, membrane potential and matrix pH are also key modulators of both F-ATPase activity and PTP. Electrophysiological experiments, after isolation and insertion of F-ATPase dimers in artificial phospholipid bilayers, showed that the pore activity matches that of PTP-MMC (Giorgio et al., 2013).

The involvement of F-ATPase dimers in PTP formation has been extended and confirmed in yeast and *Drosophila*, even if these organisms show specific differences. In yeast mitochondria the ionophore ETH129 is needed for Ca^2+^ uptake in the matrix and the PT displays a low conductance (around 300 pS). Phosphate acts as an inhibitor of PT, while CsA does not interfere with PTP. Yeast mutants lacking of subunits e and g, which are involved in dimerization, display a striking resistance to PTP opening (Carraro et al., 2014). In *Drosophila* (von Stockum et al., 2015), PTP has been initially identified as mitochondrial Ca^2+^-induced Ca^2+^ release channel (mCrC). The main differences between mCrC and mammalian PTP are: (i) absence of swelling; (ii) absence of CsA effect, since no CyPD is present in this species; (iii) sensitivity to rotenone, an inhibitor of Complex I; (iv) inhibition of mCrC by P_i_; (v) low conductance (around 53 pS) of the F-ATPase dimers in artificial bilayer.

Other research groups have also suggested that F-ATPase is involved in pore formation by the channel activity within the c-ring formed by c subunits of F_O_ (Bonora et al., 2013; Alavian et al., 2014). Nevertheless, this hypothesis is still under debate, since it does not justify the different pore size observed in bovine, yeast, and *Drosophila*, where similar c-rings are present (Bernardi et al., 2015). Finally, the possible involvement of IF_1_ in modulation of PTP through F-ATPase dimerization needs further investigations (Faccenda et al., 2013; Bernardi et al., 2015).

The presence in plants of many common components and features of F-ATPase lead us to speculate that, similarly to mammals, yeast, and *Drosophila*, PT function could be exerted by F-ATPase dimers also in such organisms.

## The Emergence of PT During Evolution

Evolution does not always proceed by adaptations. It may also develop a non-adaptive exaptation/cooptation (pre-adaptation), where the term exaptation/cooptation means a trait evolved to accomplish a specific function (or even no function), which may be then exapted/coopted to perform a novel function (or to acquire a function) (Gould and Vrba, 1982).

**FIGURE 1:**
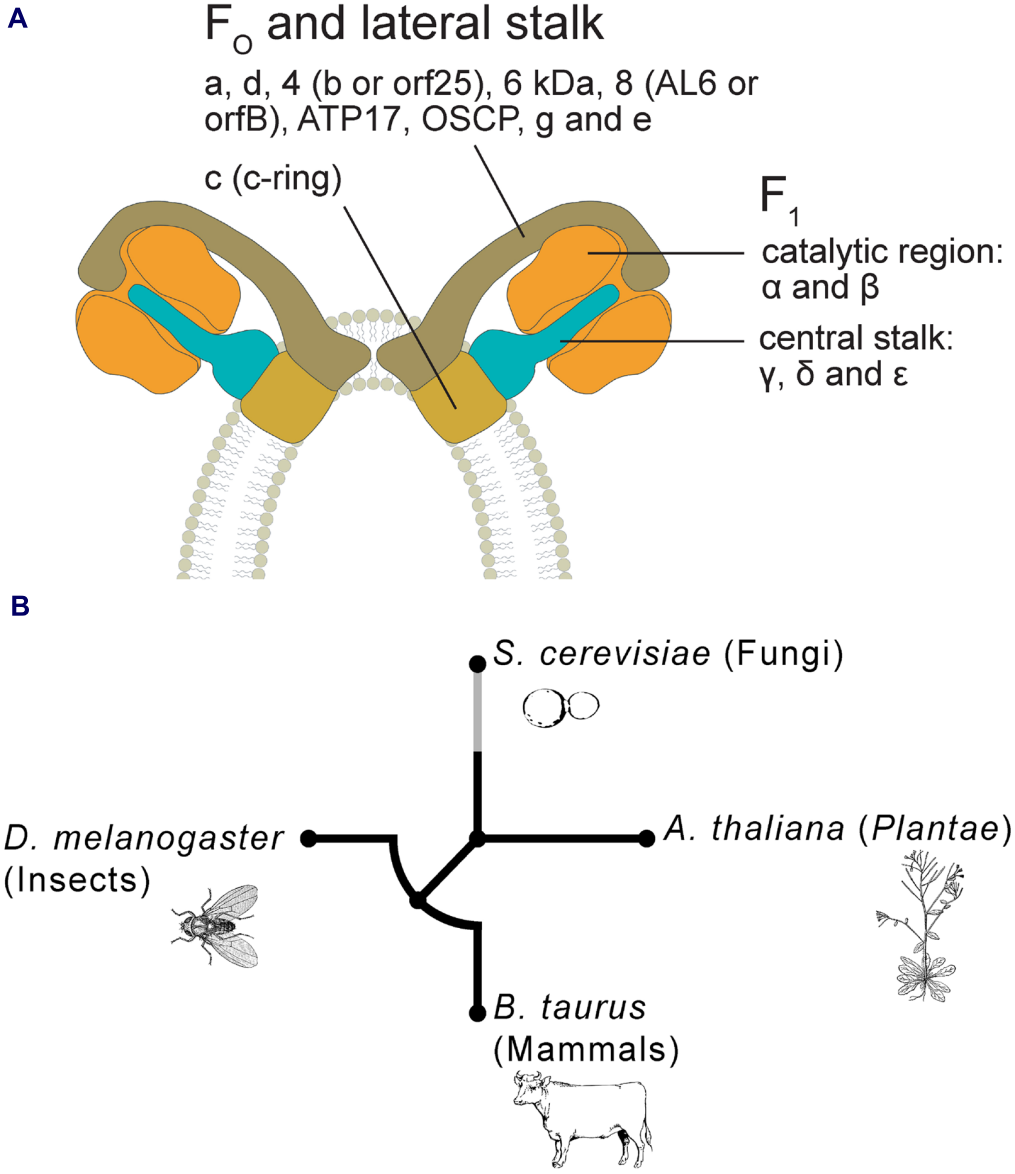
**(A)** Hypothetical model of PTP in plants, based on F-ATPase dimer formation, as proposed by Bernardi (2013b), Bonora et al. (2013), and Alavian et al. (2014). Plant F-ATPase subunits are organized on the basis of their putative correspondence to the mammalian ones. **(B)** Circular phylogenetic tree of peptide sequences of homologous subunit g of mitochondrial ATP synthase in four representative *taxa* (i.e., *Bos taurus*, *Drosophila melanogaster*, *Saccharomyces cerevisiae*, and *Arabidopsis thaliana*). Alignments of multiple amino acid sequences were performed using MUSCLE software (Edgar, 2004). Phylogenetic trees were obtained using phyML version 3.0 with the maximum-likelihood (ML) method (Guindon et al., 2010). The NCBI Reference Sequence accession codes for the g subunit are: *B. taurus* = NP_001019721; *D. melanogaster* = NP_609142; *S. cerevisiae* = NP_015345; *A. thaliana* = NP_179558. Where more isoforms were found in NCBI databases, we randomly selected only one of these sequences.

It has been suggested that the structure of PTP (as a multicomponent complex, Bernardi, 2013a) may have arisen by a mechanism of molecular exaptation, a phenomenon largely recognized at different levels of complexity (genes, proteins, organs), during evolution (Vianello et al., 2012; Barve and Wagner, 2013). The new model, involving F-ATPase dimer in PTP formation, does not contradict our previous interpretation on its origin, but rather appears to support it further. The dimer appears to be the result of a molecular exaptation/cooptation, where two monomers are assembled to perform an additional function (**Figure 1A**). In other words, F-ATPase seems to have a “Janus double face”, catalyzing the synthesis of ATP, but in some circumstances preventing such a synthesis (Bernardi et al., 2015). This dimer could even possess a “triple face”, because the dimerization induces also the curvature of the IMM.

The F-ATPase dimer is present in eukaryotes, but not in prokaryotes, because the F-ATPase of the latter is lacking of some crucial subunits (e and g) involved in dimer formation (Antoniel et al., 2014). It is thus reasonable to assume that the dimer/PTP may be arisen after the endosymbiosis between an alpha-proteobacterium and an archaeon (Martin and Müller, 1998). At the beginning, these dimers could have transferred ATP from the endosymbiont to the cytoplasm of the host cell, because the former presumably did not have ATP/ADP transporters. PTP was then maintained to dissipate the protonmotive force, thus regulating both ATP synthesis and exchanges of solutes between the cytoplasm and the mitochondrial matrix.

The presence of F-ATPase dimer has been assessed in different evolutionary divergent eukaryotes, some of which exhibit mitochondrial PT, such as ‘*Unikonts’* (*Opisthokonts*) and *Plantae* (Arpagaus et al., 2002; Giorgio et al., 2013; Carraro et al., 2014; von Stockum et al., 2015). To understand the phylogenesis of this structure/function, a cladogram has been generated by comparing the ancestral sequences of F_O_ subunit g from bovine and *Drosophila* (animals), yeast (fungi, *Ascomycetes*), and *Arabidopsis* (*Plantae*) (**Figure 1B**). The tree suggests an early differentiation at higher taxonomical levels (supergroups): *Plantae* show the highest phylogenetic distance and within the *Opisthokonts*, mammals, and insects exhibit similar distances, whereas yeast shows a higher distance. These phylogenetic patterns are consistent with the main evolutionary life tree (e.g., Keeling et al., 2005).

It has been suggested that F-ATPase shows a progressive differentiation along the main steps of evolution. In turn, some features of PTP seem to be occurred independently from changes in ATP synthase. As an example, swelling of mitochondria occurs only in bovine (Bernardi et al., 2015) and in some plants (see **Table 1**), suggesting that PTP has been differently shaped by exaptation during the evolution. Hence, exaptation leading to PT seems to have occurred in diverse contexts during life history, depending on the molecular characteristics of F-ATPase structure and the specific requirements of the respective *taxa*.

## Future Directions

The molecular nature of PTP in plants is still elusive. Further structural and functional studies are required to verify if F-ATPase dimers represent the channel associated to PT also in plants. This is needed to understand better the relationship between mitochondrial PT and PCD in plants.

## Author Contributions

MZ and AV co-supervised the manuscript and co-wrote the article. VC, EP, CP, SP, AB, and EB co-wrote the article. VDC and FB performed the phylogenetic analyses and co-wrote the article.

## Conflict of Interest Statement

The authors declare that the research was conducted in the absence of any commercial or financial relationships that could be construed as a potential conflict of interest.
